# Identification and Characterization of the Complete Genome of the TGF-β Gene Family in *Tupaia belangeri*: Expression and Function of Adipose Tissue Under Cold Acclimation Conditions

**DOI:** 10.3390/ijms26146681

**Published:** 2025-07-11

**Authors:** Lijie Du, Wanlong Zhu, Lin Zhang

**Affiliations:** 1 School of Life Sciences, Yunnan Normal University, Kunming 650500, China; d15698263058@163.com; 2 School of Basic Medical Sciences, Hubei University of Chinese Medicine, Wuhan 430065, China

**Keywords:** *Tupaia belangeri*, TGF-β, gene family, cold domestication, adipose tissue

## Abstract

The transforming growth factor beta (TGF-β) gene family is widely distributed across the animal kingdom, playing a crucial role in various cellular processes and maintaining overall health and homeostasis. The present study identified 34 TGF-β family genes based on the genome sequence in *Tupaia belangeri*, which were classified into the TGF-β, bone morphogenetic protein (BMP), growth differentiation factor (GDF), glial cell-derived neurotrophic factor (GDNF), and Activin/Inhibin subfamilies. A phylogenetic analysis revealed the evolutionary relationships among members of the TGF-β family in *T. belangeri* and their homologous genes in *Homo sapiens*, *Mus musculus*, and *Pan troglodytes*, indicating a high degree of conservation throughout evolution. A chromosomal distribution and collinearity analysis demonstrated the localization of these genes within the genome of *T. belangeri* and their collinearity with genes from other species. A gene structure and motif analysis further illustrated the conservation and diversity among TGF-β family members. A protein interaction network analysis highlighted the central roles of TGFB1, TGFB3, BMP7, and BMP2 in signal transduction. A functional enrichment analysis underscored the significance of the TGF-β signaling pathway in the biological processes of *T. belangeri*, particularly in cell proliferation, differentiation, and apoptosis. We assessed the impact of cold acclimation treatment on the expression of TGF-β family proteins in the adipose tissue (white adipose tissue [WAT] and brown adipose tissue [BAT]) of *T. belangeri* using ELISA technology, finding that protein expression levels in the experimental group were significantly higher than those of in the control group. These results suggested that cold acclimation may enhance the adaptability of *T. belangeri* to cold environments by modulating the expression of TGF-β family genes. This study offers new insights into the role of the TGF-β family in the cold acclimation adaptation of *T. belangeri*, providing a scientific foundation for future genetic improvements and strategies for cold acclimation.

## 1. Introduction

With the changing global climate, there has been a significant impact on the survival of small animals. In response to environmental changes, these animals not only adapt by altering basic physiological indicators but also modulate thermogenesis by activating or inhibiting the expression of specific thermoregulatory genes. This process assists them in maintaining their body temperature [1,2]. This is especially the case for small mammals, such as *Tupaia belangeri*, which are widely distributed around the globe. In China, this species is primarily found in Yunnan, Guizhou, southwestern Sichuan, southern Guangxi, and Hainan Island, with Yunnan being its main distribution area. The diverse climatic conditions in these regions offer a rich context for researching the ecological adaptability of *T. belangeri* [3]. Moreover, the extensive genomic data available for *T. belangeri* offers significant opportunities for studying its molecular responses to environmental changes. *T. belangeri* shares a close evolutionary relationship with primates and presents a wide array of developmental and application prospects. It can serve as an effective animal model for investigating human metabolic diseases in biomedical research [4].

It is important to note that previous studies have demonstrated the significant role of the TGF-β signaling system in the cold adaptation process of mammals. For instance, research has shown that in cold environments, the expression of specific key molecules within the TGF-β signaling pathway undergoes substantial changes. These alterations, in turn, impact the metabolic characteristics of adipose tissue, promote the development and functional enhancement of brown adipose tissue, and assist animals in better adapting to cold conditions. Furthermore, the TGF-β signaling pathway is closely associated with physiological processes such as thermoregulation and energy metabolism in animals, which are essential for their survival and adaptation in cold environments.

The transforming growth factor beta (TGF-β) gene was first identified in 1978 as a factor with cell transformation activity, isolated from the culture medium of mouse sarcoma virus cell lines [5]. Subsequently, numerous factors exhibiting transformation activity were discovered, leading to the gradual development of a vast gene family [6]. The TGF-β gene family comprises a class of multifunctional cytokines that are widely present across the animal kingdom, playing a central role in various cellular processes, including cell proliferation, differentiation, apoptosis, inflammatory responses, and autophagy [7]. The diversity and complexity of TGF-β family members enable them to play critical roles in regulating body temperature and metabolic pathways in organisms. With advancements in DNA sequencing technology, members of the TGF-β family in species such as *Homo sapiens*, *Mus musculus*, and *Pan troglodytes* have been identified; however, research on the TGF-β gene family in T. belangeri remains unexplored [8,9,10].

The current study employed bioinformatics to analyze the differential expression of genes and to investigate which of these differentially expressed genes play significant roles in temperature regulation and metabolic pathways in *T. belangeri*. The findings provide insights into gene function enrichment and the regulation of metabolic pathways, facilitating an analysis of differential gene functions related to energy metabolism. Furthermore, this research elucidates the adaptation mechanisms of *T. belangeri* to temperature fluctuations, offering valuable references for studying temperature adaptation strategies in small mammals and human metabolic diseases [11,12].

## 2. Results

### 2.1. Identification and Characterization of TGF-β Family Genes

Through HMMER (hidden Markov modeler) retrieval and subsequent BLAST (basic local alignment search tool) confirmation, 34 TGF-β family genes were identified from the complete genome of *T. belangeri*. These genes can be categorized into the TGF-β, BMP (bone morphogenetic protein), GDF (growth differentiation factor), GDNF (glial cell-derived neurotrophic factor), and Activin/Inhibin subfamilies, comprising seven genes (TGFB1, TGFB2, TGFB3, MSTN, NODAL, AHM, and LEFTY2), nine genes (BMP2, BMP3, BMP4, BMP5, BMP6, BMP7, BMP8A, BMP10, and BMP15), nine genes (GDF2, GDF3, GDF5, GDF6, GDF9, GDF10, GDF11, GDF15, and TSORFli268), four genes (PSPN, GDNF, NRTN, and ARTN), and five genes (INHA, INHBA, INHBB, INHBC, and INHBE). The analysis of the physicochemical properties of TGF-β family proteins in *T. belangeri* revealed significant variability in the number of amino acids among the TGF-β gene family members. The amino acid count for these proteins ranged from 154 (PSPN) to 598 (GDF6), while the relative molecular weight of the protein sequences varied between 22.39 kDa and 65.62 kDa, indicating a substantial range of molecular weight. The isoelectric point exhibited a range from 5.17 (BMP10) to 11.51 (ARTN). The instability coefficient varied from 40.99 (MSTN) to 80.63 (ART), and the lipophilicity coefficient ranged from 49.67 (ARTN) to 91.26 (TGFB1). The overall average hydrophilicity (GRAVY) of the TGF-β family members in *T. belangeri* were negative, suggesting that they are predominantly hydrophilic proteins (Table 1). Subcellular localization studies indicated that most TGF-β family members are primarily distributed outside the cell or on the plasma membrane, with some also localized in the nucleus, endoplasmic reticulum, Golgi apparatus, and peroxisomes (Table 1).

### 2.2. Phylogenetic Analysis

**Figure 1:**
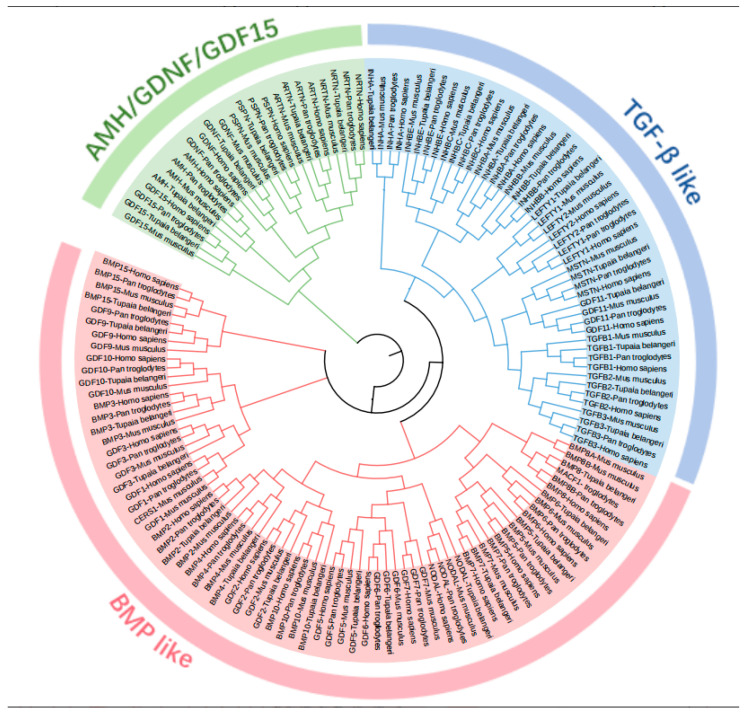
Phylogenetic tree illustrating the evolutionary relationships of the TGF-β gene family proteins among *T. belangeri*, *Homo sapiens*, *Mus musculus*, and *Pan troglodytes*. The tree is divided into three sections: AMH/GDNF/GDF15, TGF-β like, and BMP like, with different colors representing different subfamilies.

To investigate the evolutionary relationships among *T. belangeri*, humans, chimpanzees, and mice, a phylogenetic tree was constructed using the protein sequences of these four species (Figure 1). The evolutionary tree was divided into three sections: AMH/GDNF/GDF15, TGF-β like, and BMP like, encompassing a total of 144 members. Specifically, there are 47 homologous genes in the blue section and 24 homologous genes in the green section. Notably, 34 genes from *T. belangeri* cluster with members of the *H. sapiens*, *M. musculus*, and *P. troglodytes* gene families, indicating a close evolutionary relationship and suggesting potential functional similarities. The TGFB3 genes in humans and mice were positioned on similar branches, highlighting a close genetic relationship between these species. Additionally, both TGFB1 and TGFB3 exhibited branches across multiple species, demonstrating a high level of conservation among them.

### 2.3. Chromosome Distribution and Collinearity Analysis

**Figure 2:**
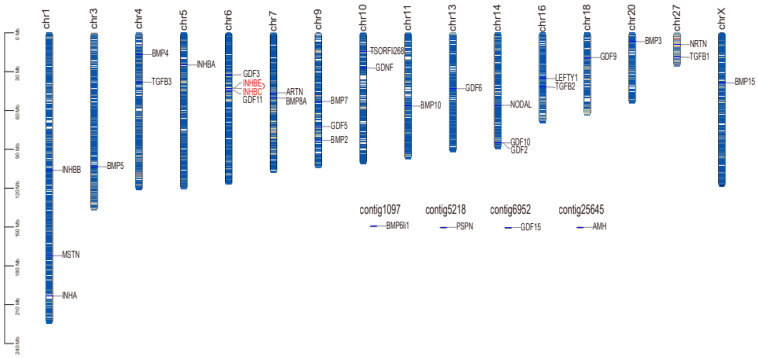
Chromosomal localization of TGF-β gene family members in *T. belangeri.* The red color in the figure represents the concatenated repeated sequence.

**Figure 3:**
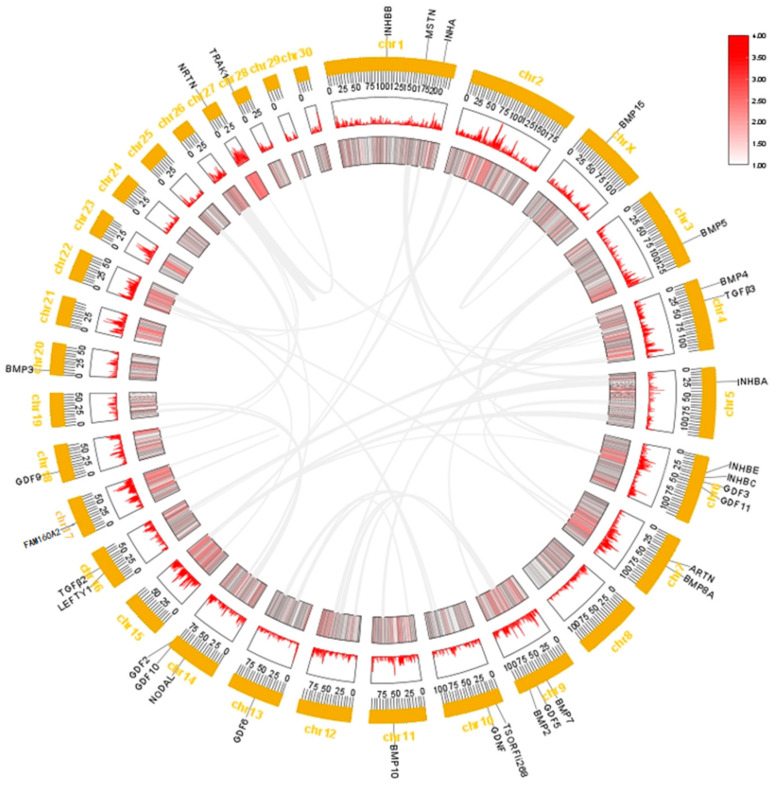
Intraspecific collinearity of the TGF-β gene family in *T. belangeri.*

**Figure 4:**
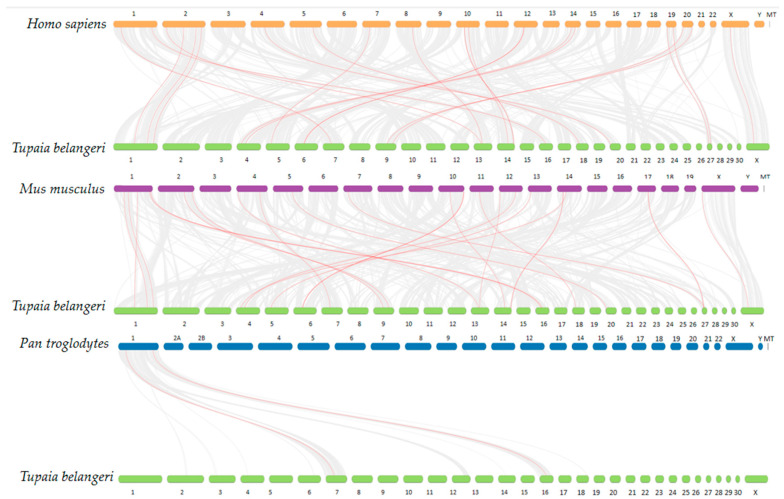
Inter-species collinearity of the TGF-β gene family among *T. belangeri*, *Homo sapiens*, *Mus musculus*, and *Pan troglodytes*.

We extracted the location information of the TGF-β gene family members from the GFF3 file of the genome of *T. belangeri* and created chromosome location maps. These genes were distributed across 16 chromosomes and four overlapping groups (Figure 2). Notably, chromosome 6 contains the highest number of TGF-β gene family members, with four identified in *T. belangeri*. Additionally, three members of the TGF-β gene family were located on chromosomes 1, 9, and 14, while two members each were found on chromosomes 4, 7, 10, 16, and 27. One member of the TGF-β gene family was located on chromosomes 3, 5, 11, 13, 18, 20, X, and the four overlapping groups. There was a tandem repeat sequence of the genes INHBE and INHBC located on chromosome 6. BMP3 exhibited similar positions and color gradients (expression levels) across multiple chromosomes, suggesting its conservation and potential critical role in bone development (Figure 3). The expression patterns of the genes INHBA, BMP4, BMP5, GDF9, GDF11, TGFB2, and NODAL were also similar across multiple chromosomes, indicating that their functions may be conserved in these regions. In the inter-species collinearity plot, numerous lines connect *H. sapiens* and *M. musculus* with *T. belangeri*, highlighting a close genetic relationship and high genetic similarity among them (Figure 4). Conversely, there were few lines connecting *P. troglodytes* and *T. belangeri*, suggesting a significant degree of genomic rearrangement between these species.

### 2.4. Gene Structure and Motif Analysis of TGF-β Gene Family

**Figure 5:**
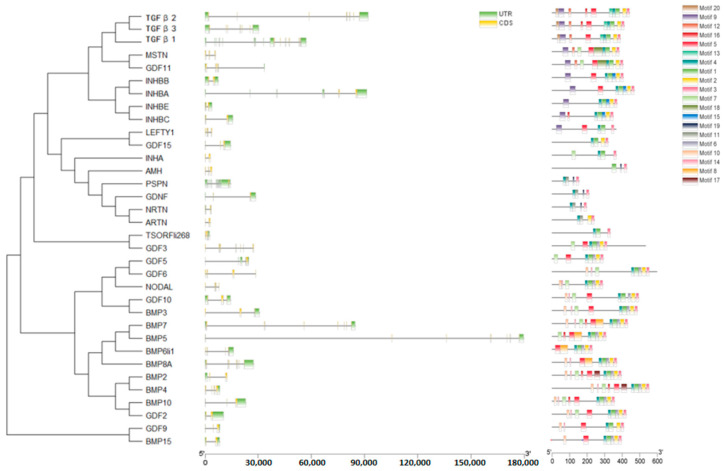
Gene structure and motif analysis of the TGF-β gene family in *T. belangeri.*

The gene structures of the TGF-β gene family members in *T. belangeri* were constructed based on the GFF3 file of *T. belangeri* genome (Figure 5). As illustrated in Figure 3, the TGF-β gene family members of *T. belangeri* exhibited a range of exon counts, with one member containing 10 exons, three members containing 8 exons, two members containing 7 exons, two members containing 5 exons, four members containing 4 exons, eight members containing 3 exons, and fourteen members containing 2 exons. Notably, the predominant number of exons among the TGF-β gene family members was two. All TGF-β gene family members in *T. belangeri* contain introns; however, the number of introns varies significantly among different members, with GDNF exhibiting the highest intron count. This variation in the number of exons and introns among the TGF-β gene family members in *T. belangeri* highlighted the diversity within the gene family proteins in *T. belangeri*. A motif analysis was performed on the TGF-β gene family protein sequence of *T. belangeri*, with a conserved motif count of 20. From Figure 5, it can be seen that all 34 TGF-β gene family protein sequences of *T. belangeri* contain 14 conserved motifs. The number of conserved motifs in all 34 TGF-β gene family protein sequences of *T. belangeri* was greater than or equal to 3, with BMP2 and BMP4 containing the same number of conserved motifs, 11, which indicated that most of the TGF-β gene family proteins in *T. belangeri* have high conservation.

### 2.5. Protein Interaction Analysis Network of TGF-β Family Proteins

**Figure 6:**
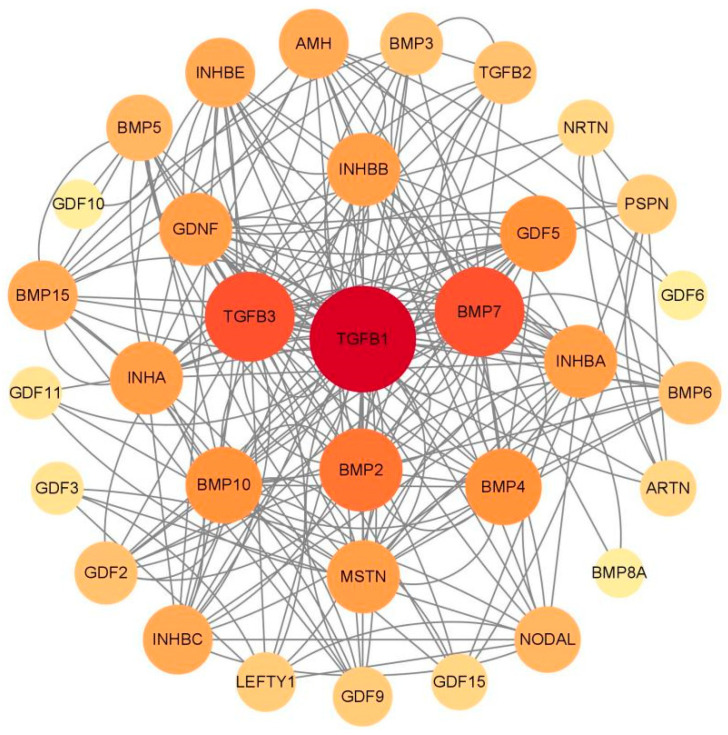
Protein interaction network analysis of TGF-β family genes in *T. belangeri*. Node colors represent the degree of connectivity within the network, with darker shades indicating higher connectivity. The central node, TGFβ1, is highlighted in dark red to denote its significant role in the network. Other TGF-β family members are shown in varying shades of orange, reflecting their interaction strength with TGFβ1 and other proteins. Lines represent interactions between proteins.

In protein network diagrams, each node corresponds to a distinct protein. The size of these nodes is indicative of the protein’s degree, which is to say, the quantity of interactions it has with other proteins. Edges in the diagram signify the interactions between proteins. Within the TGF-β family, the proteins that exhibited the most extensive connectivity, as depicted in a tree protein interaction network (Figure 6), are TGFB1, TGFB3, BMP7, and BMP2. Additionally, there were interactions among other members of the TGF-β family. Notably, TGFB1, TGFB3, BMP7, and BMP2 emerged as central nodes within the network, underscoring their pivotal role in the TGF-β signaling cascade.

### 2.6. Functional Enrichment Analysis

**Figure 7:**
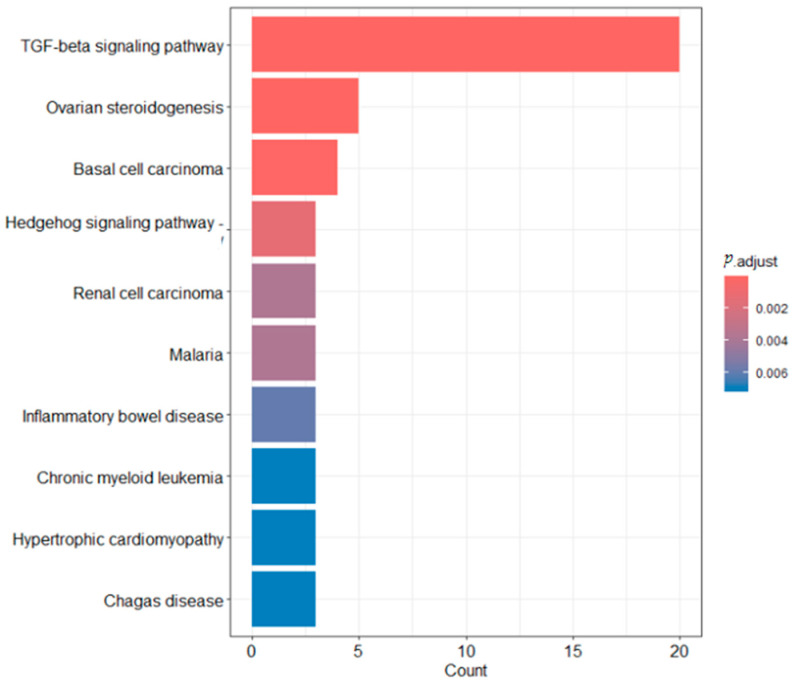
GO enrichment analysis of TGF-β family members in *T. belangeri.*

**Figure 8:**
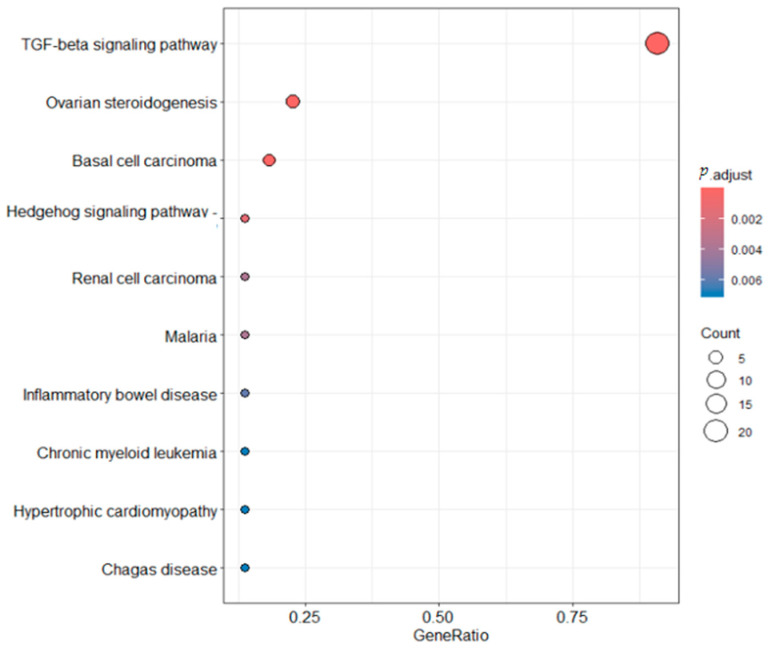
KEGG enrichment analysis of TGF-β family members in *T. belangeri.*

To elucidate the biological roles of the TGF-β gene family in *T. belangeri*, ClusterProfiler was employed for a functional enrichment analysis of this gene family (Figure 7). The Gene Ontology (GO) analysis revealed that the TGF-β signaling pathway, also known as the transforming growth factor-beta signaling pathway, was the most prominent in the graph, with a count approaching 20. This suggested a high prevalence of genes or proteins associated with the TGF-β signaling pathway within the analyzed samples. The pathway is highlighted in the darkest red, indicating an extremely low *p*-value, well below 0.002, which signifies a highly significant correlation with the research findings. Even after adjustments for multiple comparisons, this correlation remains robust, underscoring the pivotal role of the TGF-β signaling pathway in various biological processes, including cell proliferation, differentiation, apoptosis, and immune regulation. Both ovarian steroidogenesis and basal cell carcinoma pathways showed counts around 5 and are depicted in a deep red hue, denoting their considerable significance in research. In contrast, the significance of other pathways was comparatively low. The KEGG results (Figure 8) indicated that the GeneRatio for the TGF-β signaling pathway was nearly 1, reflecting a substantial involvement of genes in this pathway within the study. The *p*.adjust is the most red, representing the smallest *p*-value and the greatest statistical significance. The count was substantial. Conversely, the GeneRatio for the ovarian steroidogenesis and basal cell carcinoma pathways was relatively low, yet they still command a certain presence. These pathways are marked in a dark red color with a small *p*-value, signifying high statistical significance. Other pathways exhibited a very low GeneRatio, minimal counts, and are thus of lesser consequence.

### 2.7. Analysis of Differentially Expressed Genes

**Figure 9:**
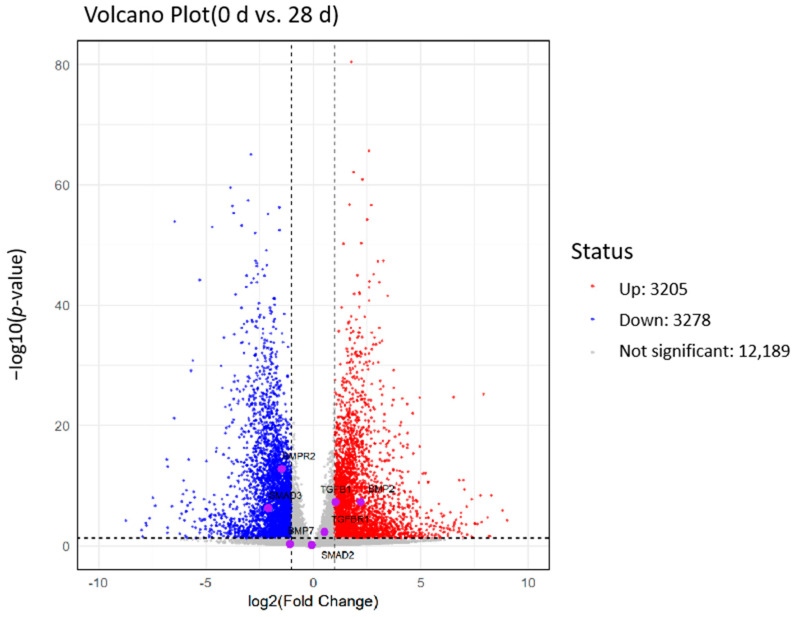
Volcanic map of differentially expressed genes in *T. belangeri.* The horizontal axis represents the log2 fold change in gene expression, while the vertical axis depicts the negative logarithm of the *p*-value (-log10 *p*-value), which is used to assess statistical significance. The label “0 d vs. 28 d” indicates a comparison of gene expression levels between day 0 (initial condition) and day 28 (after 28 days of cold acclimation treatment).

Figure 9 illustrates the changes in gene expression between day 0 (0 d) and day 28 (28 d). The horizontal axis of the figure represents the log2 fold change in gene expression, while the vertical axis depicts the negative logarithm of the *p*-value (−log10 *p*-value), which is used to assess statistical significance. The red dots in the figure indicate upregulated genes (3205), the blue dots represent downregulated genes (3278), and the gray dots signify genes with no significant changes (12,189). Most gene expression changes were not statistically significant and were concentrated in the central region of the graph. The distribution of upregulated and downregulated genes appeared roughly symmetrical, suggesting a trend of both upregulation and downregulation of gene expression between these two time points. This symmetry may reflect the organisms’ responses to environmental changes or internal regulatory mechanisms at different time points. Additionally, BMPR2 and SMAD3 were significantly downregulated in the region depicted in the figure, indicating that the expression levels of these genes were considerably lower at day 28 compared to day 0. Conversely, BMP2 showed upregulation, which contrasted with its receptor BMPR2, potentially indicating the activation of specific inhibitory signaling pathways.

### 2.8. ELISA Detects Protein Content

**Figure 10:**
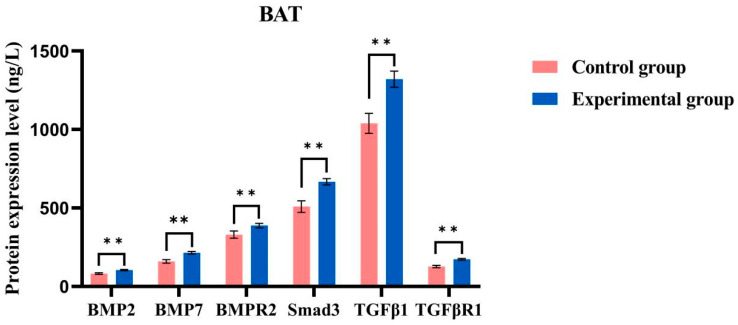
Analysis of protein expression levels in the BAT experimental group and control group of *T. belangeri.* The symbol “**” indicates statistical significance compared to the control group (*p* < 0.0001).

**Figure 11:**
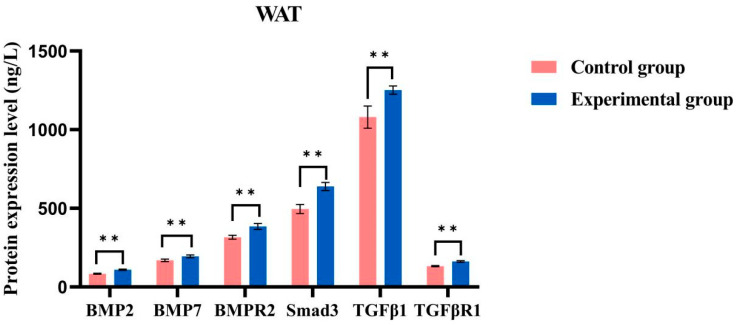
Analysis of protein expression levels in the WAT experimental group and control group of *T. belangeri.* The symbol “**” indicates statistical significance compared to the control group (*p* < 0.0001).

The experimental group with brown adipose tissue (BAT) demonstrated higher expression levels of all tested proteins (BMP7, BMP2, BMPR2, Smad3, TGF-β1, and TGF-βR1) compared to the control group (Figure 10). Each experiment was conducted in triplicate, and the results are presented as the mean ± standard deviation (SD) from three independent experiments. The sample size for each group was *n* = 6, which ensured adequate statistical power to detect significant differences. The results of the independent sample t-test revealed that the difference between the experimental and control groups was highly significant (*p* < 0.0001), with the average expression levels in the experimental group being substantially greater than those in the control group. These findings suggest that cold acclimation positively influences the expression of these genes. Similarly, the expression levels of all tested genes in the white adipose tissue (WAT) experimental group were generally higher than those in the WAT control group (Figure 11), and these differences were statistically significant (*p* < 0.0001), indicating that cold acclimation treatment significantly affects gene expression in this tissue as well.

## 3. Discussion

As an important experimental animal model, the adaptation mechanisms of *T. belangeri* to cold environments have consistently been a research hotspot. The TGF-β gene family plays a crucial role in various biological processes, including cell proliferation, differentiation, apoptosis, and immune regulation, and is closely associated with the metabolic characteristics of adipose tissue. The aim of this study was to investigate the genome-wide characteristics of the TGF-β gene family and its expression changes in different adipose tissues of *T. belangeri* under cold acclimation conditions in order to elucidate its adaptation mechanisms to cold environments. Through a comprehensive analysis of the genome of *T. belangeri*, we identified 34 members of the TGF-β family, which we categorized into five subfamilies: TGF-β, BMP, GDF, GDNF, and Activin/Inhibin. This classification indicated that the TGF-β family exhibits significant diversity and complexity within the genome of *T. belangeri* [13]. These gene family members exhibited significant variability in amino acid composition, molecular weight, the isoelectric point, the instability coefficient, and the lipid solubility coefficient [14]. This variability may be closely related to their distinct roles in the biological functions of *T. belangeri*. Further analysis revealed that the protein hydrophilicity, indicated by negative GRAVY values, of TGF-β family members in *T. belangeri* suggested that these proteins were likely to function in aqueous environments. This characteristic may be associated with their roles in various biological processes within cells [15]. The subcellular localization analysis demonstrated the distribution of TGF-β family members within the cells of *T. belangeri*, with the majority located extracellularly or on the plasma membrane. This distribution may be linked to their role in intercellular signaling [16]. Additionally, the localization of some members in the nucleus, endoplasmic reticulum, Golgi apparatus, and peroxisome indicates that TGF-β family proteins may be involved in a range of intracellular processes, from gene expression regulation to the maintenance of cell structure [17].

The TGF-β family members of *T. belangeri* are clustered with homologous genes from humans, mice, and chimpanzees, indicating their high conservation throughout evolution [18]. The conservation of the TGF-β gene family across species suggested that the mechanisms underlying cold adaptation in *T. belangeri* may share similarities with those in other small mammals. The findings of this study regarding the role of the TGF-β gene family in the cold acclimation of *T. belangeri* provide valuable insights into the broader ecological adaptations of small mammals to varying environmental temperatures. By elucidating the specific roles of TGF-β family members in *T. belangeri*, our research contributed to a more comprehensive understanding of the evolutionary strategies employed by small mammals to survive in cold environments. Additionally, the TGF-β genes in the genome of *T. belangeri* were unevenly distributed across 16 chromosomes, with the number of genes on each chromosome ranging from one to four. There was no clear correlation between the chromosomal locations of TGF-β genes and the sequence similarity among these genes, suggesting that most of them are relatively primitive [19]. This uneven distribution implied that genetic variability is greater during evolution, potentially due to the loss and acquisition of genes. Chromosomal distribution and collinearity analyses further elucidated the similarities and differences in genome structure between *T. belangeri* and these species [20]. The members of the TGF-β gene family in *T. belangeri* possessed between 2 to 10 exons, and this wide range of exon numbers reflected the structural diversity of TGF-β family members [21]. This structural diversity may be closely linked to their functional diversity in various biological processes. Notably, the GDNF gene contained the most introns, which may be associated with its specific biological functions. The motif analysis of the TGF-β gene family protein sequences in *T. belangeri* revealed that most of the 34 members contained at least 14 conserved motifs. This finding suggested that these genes have retained key functional domains throughout evolution [22]. Notably, the BMP2 and BMP4 genes, which possess 11 conserved motifs, may be essential for their structural stability and functionality. Variations in the number of exons and introns could influence gene expression regulation and the post-translational modification of proteins. Additionally, the presence of conserved motifs highlighted critical regions of protein function that remain consistent across different species, which were vital for preserving the fundamental roles of proteins. The protein interaction network analysis further underscores the central roles of TGFB1, TGFB3, BMP7, and BMP2 in signal transduction, suggesting that these proteins may serve as key nodes in regulating biological processes in *T. belangeri* [23,24].

The GO analysis results indicated that the TGF-β signaling pathway exhibited the highest count among all analyzed pathways. This finding suggested that the expression frequency of genes or proteins associated with TGF-β signaling was abnormally elevated in the study samples. Furthermore, the *p*.adjust value for this pathway was exceptionally low, significantly below 0.002, which remained statistically significant even after correction for multiple comparisons. This underscores the pivotal role of the TGF-β signaling pathway in a biological context, particularly in essential processes such as cell proliferation, differentiation, apoptosis, and immune regulation [25]. To put it simply, the GO enrichment analysis highlighted the TGF-β signaling pathway as a crucial component in the biological processes of *T. belangeri.* This pathway was involved in a wide range of essential cellular activities, including cell growth, differentiation, and apoptosis. The prominence of this pathway in our analysis indicated that it is particularly active and significant in the context of cold acclimation. In other words, when *T. belangeri* was exposed to cold conditions, the TGF-β signaling pathway was likely a major factor in the animal’s molecular adaptation and response. The KEGG analysis further demonstrated that the Gene Ratio of the TGF-β signaling pathway is close to 1, indicating that this pathway encompasses the vast majority of the genes studied. This finding further confirmed the central role of the TGF-β signaling pathway in the biological functions of *T. belangeri*. Additionally, the ovarian steroidogenesis and basal cell carcinoma pathways also exhibited high Gene Ratios, which may be associated with the significant role of the TGF-β signaling pathway in regulating these biological processes [26,27]. After studying the TGF-β signaling pathway, it was determined that the signaling of the TGF-β receptor complex was initiated by TGF-β. TGF-β (TGFB1) is secreted as a homodimer, which binds to TGF-β receptor II (TGFBR2), inducing its dimerization and forming a stable heterotetrameric complex with the TGF-β receptor I homodimer (TGFBR1). The phosphorylation of TGFBR1, mediated by TGFBR2, triggers the internalization of the heterotetrameric TGF-β receptor complex (TGFBR) into clathrin-coated endocytic vesicles and recruits cytoplasmic SMAD2 and SMAD3, which function as R-SMADs of the TGF-β receptor complex. TGFBR1 can phosphorylate SMAD2 and SMAD3, facilitating their binding with SMAD4 [13]. In the nucleus, SMAD2/3/4 heterotrimers bind to target DNA elements and work synergistically with other transcription factors to regulate gene expression involved in cell differentiation [25].

Under cold acclimation conditions, significant changes were observed in the expression of TGF-β family genes in the adipose tissue of *T. belangeri*. BMP2 positively influences bone health and strength, particularly in response to physiological stress in cold environments [28]. By promoting the activation of BAT and enhancing thermogenesis, BMP2 assists *T. belangeri* in maintaining its body temperature in cold environments. Therefore, based on the experimental results and research on metabolic pathways, we focused on the expression levels of genes such as BMP7, BMP2, BMPR2, Smad3, TGF-β1, and TGF-βR1 under cold acclimation conditions [29]. In the experimental group, the expression levels of these six genes in BAT were significantly higher than those in the control group. This suggested that the upregulation of gene expression under cold acclimation conditions may be associated with an increase in BAT, which plays a critical role in energy expenditure and heat production [30]. However, due to the inhibitory effects of the TGF-β signaling pathway, the expression levels of BMP2 and BMP7 and their receptor BMPR2 were low. The differential gene analysis, however, indicated that BMP2 was upregulated while BMPR2 was downregulated. It is known that type II receptors can form dimer complexes even in the absence of BMP2 [31]. Similarly, TGFBR3 antagonizes BMP2 signaling by binding to ACVR2A. TGFBR3 competes with BMP2 for ACVR2A, thereby inhibiting BMP2 signaling [32] and resulting in the lowest expression level of BMP2. Among them, BMP7 promotes the expression of the brown fat marker gene UCP1 during the differentiation of brown adipocytes. The overexpression of BMP7 significantly increases the weight of brown fat in mice [33]. The bone morphogenetic protein BMP7, also known as the brown adipose tissue determinant, stimulates the formation of active SMAD trimers, with TGFB1, TGFB3, BMP7, and BMP2 at the core of these protein interactions [34]. Under winter or low-temperature conditions, the expression level of BMP7 in *T. belangeri* is upregulated, promoting the formation of brown adipocytes and increasing white adipose tissue to compensate for insufficient heat production and adapt to the cold winter environment. In the cold treatment group, the gene expression levels in the WAT were generally higher than those in the control group. This difference may be associated with the transformation of WAT into BAT [28]. Specifically, the upregulation of BMP7 and BMP2, while modest, may reflect a fine-tuning mechanism in the activation of BAT and the enhancement of thermogenesis. This fine-tuning could be crucial for maintaining thermal homeostasis in *T. belangeri* under cold conditions, as even slight increases in thermogenic capacity may be beneficial for survival. Furthermore, the observed changes in protein expression might represent early responses to cold stress, which could be amplified through downstream signaling pathways and cellular processes. Additional studies are necessary to elucidate the precise biological mechanisms underlying these subtle yet significant changes and their role in the overall cold adaptation strategy of *T. belangeri*. BAT is essential for adapting to cold environments due to its high metabolic activity and thermogenic capacity. Cold acclimation significantly influences the expression of TGF-β family genes, which may be linked to the adaptability of *T. belangeri* to cold climates [35]. The TGF-β family of genes plays a crucial role in cell proliferation, differentiation, and apoptosis. Changes in their expression may promote the metabolic reprogramming of adipocytes, thereby enhancing the cold tolerance of *T. belangeri* [36]. Alterations in the expression of TGF-β family genes can influence the metabolic characteristics of adipose tissue by regulating the differentiation and function of adipocytes. For instance, BMP2 and BMPR2 are essential for bone formation and adipocyte differentiation, while Smad3, a key effector molecule in the TGF-β signaling pathway, may regulate adipocyte responses [30]. The TGF-β family of genes may also facilitate the transformation between different types of adipose tissue by affecting the proliferation, differentiation, and apoptosis of adipocytes, as well as modulating the metabolic activity of adipose tissue. The upregulation of these genes may promote the conversion of WAT to BAT, thereby enhancing the cold adaptation capabilities of *T. belangeri* [35].

## 4. Material and Method

### 4.1. Identification and Characterization of TGF-β Family Genes

The genome sequence, protein sequence, and annotation files of *T. belangeri* were sourced from the Tree Shrew Database at the Kunming Institute of Zoology, Chinese Academy of Sciences (http://www.treeshrewdb.org/ [accessed on 10 March 2024]). Information regarding the TGF-β domain (PF00019) was downloaded from the Pfam database (http://pfam.xfam.org/ [accessed on 10 March 2024]) and identified using HMMER software (vrsion 3.4) [37]. Further confirmation was conducted using the BLAST tool available at NCBI (https://blast.ncbi.nlm.nih.gov/Blast.cgi [accessed on 10 March 2024]) and the UniProt database (https://www.uniprot.org/ [accessed on 11 Mar 2024]). An online analysis of the identified TGF-β gene family member proteins in *T. belangeri* was performed using ExPASy (http://www.expasy.org [accessed on 11 Mar 2024]), which will assess amino acid quantity, relative molecular weight, instability coefficient, isoelectric point, lipophilicity coefficient, and hydrophilicity. Additionally, subcellular localization prediction analysis of these protein sequences was conducted using WoLF PSORT Prediction (https://wolfpsort.hgc.jp/ [accessed on 11 Mar 2024]).

### 4.2. Phylogenetic Analysis

A phylogenetic tree was constructed using MEGA7 software, and a neighbor-joining (NJ) analysis was performed on the TGF-β gene family of *H. sapiens* (GCA_000001405.29), *M. musculus* (GCA_000001635.9), *P. troglodytes* (GCA_000001515.5), and *T. belangeri*. The neighbor-joining method was employed with the bootstrap validation parameter set to 1000, while all other values remained at their default settings [38]. After completing the construction, the online software Evolview (https://evolgenius.info/evolview-v2/# [accessed on 13 March 2024]) was utilized to modify the evolutionary tree using login credentials [39].

### 4.3. Chromosome Distribution and Collinearity Analysis

The coding sequence (CDS) of the TGF-β gene was compared with the corresponding genomic DNA sequence using TBtools (version 2.303) (https://github.com/CJ-Chen/TBtools-II [accessed on 14 March 2024]). The gene structure was visualized based on chromosomes [40]. The complete genome sequences, gene feature format (GFF) files, and annotation files were downloaded for *H. sapiens*, *M. musculus*, and *P. troglodytes* from the Ensembl database to detect collinearity between *T. belangeri* and various species [41]. By utilizing TBtools to process the data, chromosome length files, collinearity files, and gene position files were generated. Subsequently, the One Step MCScanX ultra-fast plugin in TBtools was employed for an intra-species and inter-species collinearity visualization analysis.

### 4.4. Gene Structure and Motif Analysis of TGF-β Gene Family

Using MEME (https://meme-suite.org/meme/ [accessed on 14 March 2024]), the conserved motifs of the TGF-β gene family members in *T. belangeri* were predicted, setting the number of motifs to 20 while keeping the remaining parameters at their default values. Additionally, TBtools was utilized to create a schematic diagram illustrating the gene structure and conserved sequences of the TGF-β gene family members in *T. belangeri* [42].

### 4.5. Protein Interaction Analysis Network of TGF-β Family Proteins

The identified TGF-β family proteins were uploaded to the STRING database (https://string-db.org/ [accessed on 15 March 2024]) (STRING version 11.5), and Homo sapiens was selected as the organism to analyze the interactions among the family proteins using the default settings. The composite score for protein interactions was derived from STRING data, which includes factors such as homology, co-expression, and database annotations [43].

### 4.6. Functional Enrichment Analysis

To understand the biological processes and molecular functions associated with TGF-β family gene expression in *T. belangeri*, we conducted Gene Ontology (GO) enrichment and Kyoto Encyclopedia of Genes and Genomes (KEGG) enrichment analyses on the identified related genes. We prepared a list of differentially expressed genes, which includes gene identifiers such as IDs and gene symbols. Additionally, we compiled a background gene set that encompasses all known genes of the analyzed species, which serves as the basis for the enrichment analysis. Annotation files were also created to link genes to their corresponding GO terms or KEGG pathways [44,45]. GO and KEGG enrichment analyses were performed using the ClusterProfiler package, with results corrected through multiple testing methods, and visualizations were generated using the enrichplot package [46].

### 4.7. Analysis of Differentially Expressed Genes

RNA sequencing (RNA-seq) generated gene expression data, and statistical methods such as the *t*-test, ANOVA, DESeq2, and edgeR were employed to calculate the expression differences of each gene between two conditions (e.g., day 0 and day 28), resulting in log2 fold changes and *p*-values. Genes that were significantly upregulated or downregulated were selected based on a predetermined threshold (*p* < 0.05). Finally, the ggplot2 package in R was utilized to create volcano plots [47].

### 4.8. ELISA Detects Protein Content

This study utilized healthy adult *T. belangeri* as experimental subjects, which were housed in the animal room of the School of Life Sciences at Yunnan Normal University and acclimatized to room temperature for one month. The average weight of the subjects was 115.36 ± 3.65 g. Based on their weight, the shrews were divided into two groups: the control group (*n* = 6, temperature 25 ± 1 °C) and the cold acclimation group (*n* = 6, temperature 5 ± 1 °C). Both groups were provided with moderate lighting, water, and ad libitum access to food, and they underwent acclimatization for 28 days. Following the acclimatization period, the shrews were euthanized using CO_2_, and WAT and BAT were quickly collected from the scapulae. After accurately excising the required tissues, the samples were rinsed briefly in phosphate-buffered saline (PBS) to remove blood stains and debris. The tissues were then fixed in 4% paraformaldehyde for 24 h at 4 °C. The experimental samples were then clamped with tweezers and cooled in liquid nitrogen for 15 min. The wrapped tissue samples were loaded into prepared cryovials and promptly transferred to a −80 °C freezer for storage. A total of 0.5 g of animal tissue was collected, with a backup sample retained. Dry ice was employed for low-temperature transport to prevent repeated freezing and thawing of the samples, which could lead to a loss of biological activity. The samples were sent to Kunming Baika Biotechnology Co., Ltd., where an enzyme-linked immunosorbent assay (ELISA) was performed to detect the protein content in WAT and BAT, specifically for BMP2, BMP7, BMPR2, phosphorylated Smad2/Smad3, TGF-β1, and TGF-βR1 in *T. belangeri* [48].

## 5. Conclusions

This study thoroughly investigated the genome-wide characteristics of the TGF-β gene family and its expression patterns in various adipose tissues of *T. belangeri* under cold acclimation. By integrating bioinformatics tools with experimental techniques, we successfully identified 34 members of the TGF-β family and elucidated their distribution within the genome of *T. belangeri*, as well as their collinearity with other species. The research findings indicated that the distribution of TGF-β family members in the genome of *T. belangeri* was diverse and exhibited uneven distribution across different chromosomes. The phylogenetic analysis and chromosome distribution analysis further confirmed the conservation of these genes throughout evolution, as well as their collinearity among different species. Additionally, the gene structure and motif analysis revealed both the structural conservation and diversity of TGF-β family members. The functional enrichment analysis underscored the pivotal role of the TGF-β signaling pathway in the biological processes of *T. belangeri*, particularly in critical processes such as cell proliferation, differentiation, and apoptosis. The protein interaction network analysis highlighted the significant roles of TGFB1, TGFB3, BMP7, and BMP2 in signal transduction. The results from ELISA experiments further confirmed that cold acclimation treatment significantly increases the expression levels of TGF-β family proteins in adipose tissue, which may be associated with the enhanced adaptability of *T. belangeri* to cold environments. In conclusion, this study provided new insights into the role of the TGF-β family in the cold acclimation adaptation of *T. belangeri* and established a scientific basis for future genetic improvement and cold acclimation strategies. By exploring the functions and regulatory networks of these genes, we can gain a more comprehensive understanding of the adaptation mechanisms of *T. belangeri* to cold environments. Future research should further investigate the specific molecular mechanisms of TGF-β family members in the cold domestication process of *T. belangeri*, as well as their interactions with other signaling pathways in the regulation of the biological functions of this species.

## Figures and Tables

**Table 1:** Physical and chemical properties of the TGF-β gene family proteins in *T. belangeri.*

Gene Name	Protein Serial Number	Number of Amino Acids	Relative Molecular Weight of Protein/kDa	Isoelectric Point	Instability Coefficient	Overall Average Hydrophilicity	Subcellular Localization
TGFB2	TSDBTID.10930.1	442	50,504.96	8.74	52.37	−0.393	P; N
TGFB3	TSDBTID.22079.2	412	47,353.42	8.56	49.70	−0.477	PM; P; G
TGFB1	TSDBTID.1119.1	390	44,505.37	8.92	53.05	−0.357	P; PM
MSTN	TSDBTID.4722.1	383	43,653.21	6.67	40.99	−0.399	C;P; M
GDF11	TSDBTID.24154.1	405	44,964.62	8.18	61.51	−0.299	PM; ER; P
INHBB	TSDBTID.4539.1	407	45,061.52	8.35	58.22	−0.288	P; PM
INHBA	TSDBTID.22580.1	469	52,597.32	9.04	64.26	−0.598	N; C; P
INHBE	TSDBTID.24129.1	371	40,672.71	9.28	56.76	−0.295	PM; N
INHBC	TSDBTID.24130.1	349	37,820.47	8.28	49.41	0.010	P; PM
LEFTY1	TSDBTID.10569.1	365	40,842.85	8.61	54.99	−0.302	P; PM
GDF15	TSDBTID.27506.2	320	35,836.12	9.72	55.59	−0.361	P; N; PM
INHA	TSDBTID.4074.1	366	39,189.08	8.02	64.25	−0.063	P; PM
AMH	TSDBTID.26794.1	426	46,391.31	9.00	53.59	−0.299	N; C
PSPN	TSDBTID.2357.1	154	16,218.64	9.23	42.57	−0.106	PM
GDNF	TSDBTID.6036.1	211	23,722.16	9.25	63.19	−0.560	P
NRTN	TSDBTID.18517.1	197	22,393.00	11.12	72.88	−0.436	P
ARTN	TSDBTID.25276.1	242	25,936.37	11.51	80.63	−0.846	N
TSORFli2	TSDBTID.5994.1	332	37,772.67	6.21	58.98	−0.051	P
GDF3	TSDBTID.1893.1	533	60,304.47	9.36	51.68	−0.282	P
GDF5	TSDBTID.5249.1	292	33,188.20	9.35	43.17	−0.461	N; C
GDF6	TSDBTID.8166.1	598	65,620.63	9.43	51.31	−0.585	N; C; M
NODAL	TSDBTID.8844.1	289	33,063.76	6.55	60.36	−0.401	P; PERO
GDF10	TSDBTID.8961.2	494	54,332.31	9.44	55.13	−0.455	P; ER
BMP3	TSDBTID.14860.1	487	55,013.28	9.72	69.46	−0.567	P; M
BMP7	TSDBTID.5163.1	431	49,340.96	7.35	54.47	−0.403	P; M
BMP5	TSDBTID.20495.2	308	35,007.68	9.04	52.41	−0.511	N; C
BMP6	TSDBTID.26352.1	229	25,826.29	8.47	53.87	−0.313	P
BMP8A	TSDBTID.24686.2	369	40,367.58	9.49	69.48	−0.386	P
BMP2	TSDBTID.5339.1	395	44,645.81	9.12	54.95	−0.495	P; ER
BMP4	TSDBTID.21977.2	553	62,888.18	9.94	70.04	−0.820	N
BMP10	TSDBTID.6702.2	356	40,694.94	5.17	47.32	−0.546	N; C
GDF2	TSDBTID.8962.1	423	46,732.97	7.18	49.32	−0.362	P
GDF9	TSDBTID.12441.1	409	46,828.30	8.97	57.75	−0.512	N
BMP15	TSDBTID.19556.1	396	45,786.83	9.27	55.64	−0.393	PM; PERO

Note. PM: plasma membrane; P: periplasm; N: nucleus; ER: endoplasmic reticulum; G: golgiosome; M: mitochondrion; PERO: peroxisome; C: cytoplasm.

## Data Availability

Genomics analysis data for this submission can be found online at http://www.treeshrewdb.org/ and physiological data at https://doi.org/10.6084/m9.figshare.28876424 (accessed on 8 July 2025).
